# Adipose‐derived stromal cells for nonhealing wounds: Emerging opportunities and challenges

**DOI:** 10.1002/med.21789

**Published:** 2021-02-01

**Authors:** Milena Deptuła, Agnieszka Brzezicka, Aneta Skoniecka, Jacek Zieliński, Michał Pikuła

**Affiliations:** ^1^ Laboratory of Tissue Engineering and Regenerative Medicine, Department of Embryology Medical University of Gdansk Gdańsk Poland; ^2^ Department of Plastic Surgery Medical University of Gdansk Gdańsk Poland; ^3^ Department of Embryology, Faculty of Medicine Medical University of Gdansk Gdańsk Poland; ^4^ Department of Oncologic Surgery Medical University of Gdansk Gdańsk Poland

**Keywords:** adipose‐derived stromal cells, chronic wounds, diabetic ulcers, fat transfer, oncological wounds, SVF, wound healing

## Abstract

Wound healing complications affect thousands of people each year, thus constituting a profound economic and medical burden. Chronic wounds are a highly complex problem that usually affects elderly patients as well as patients with comorbidities such as diabetes, cancer (surgery, radiotherapy/chemotherapy) or autoimmune diseases. Currently available methods of their treatment are not fully effective, so new solutions are constantly being sought. Cell‐based therapies seem to have great potential for use in stimulating wound healing. In recent years, much effort has been focused on characterizing of adipose‐derived mesenchymal stromal cells (AD‐MSCs) and evaluating their clinical use in regenerative medicine and other medical fields. These cells are easily obtained in large amounts from adipose tissue and show a high proregenerative potential, mainly through paracrine activities.

In this review, the process of healing acute and nonhealing (chronic) wounds is detailed, with a special attention paid to the wounds of patients with diabetes and cancer. In addition, the methods and technical aspects of AD‐MSCs isolation, culture and transplantation in chronic wounds are described, and the characteristics, genetic stability and role of AD‐MSCs in wound healing are also summarized. The biological properties of AD‐MSCs isolated from subcutaneous and visceral adipose tissue are compared. Additionally, methods to increase their therapeutic potential as well as factors that may affect their biological functions are summarized. Finally, their therapeutic potential in the treatment of diabetic and oncological wounds is also discussed.

## 1 INTRODUCTION

**Figure 1:**
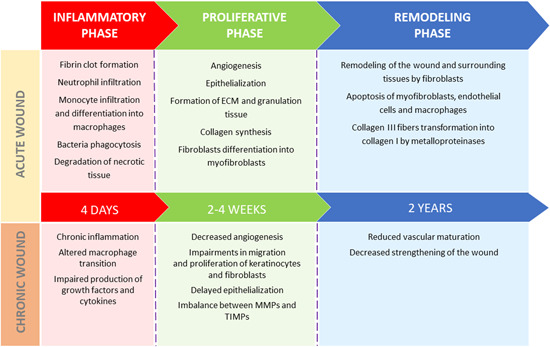
Wound healing process in acute and chronic wounds [Color figure can be viewed at wileyonlinelibrary.com]

Wound healing is a complex process consisting of three main overlapping stages: the inflammatory phase, proliferative phase, and remodeling phase, which occur in temporal sequence (Figure 1). It is regulated by different cell types (e.g., keratinocytes, fibroblasts, stem cells, and immune cells), cytokines, growth factors, and extracellular matrix components (ECM).^1^ The inflammatory phase usually lasts for the first 4 days after injury and begins with the formation of a fibrin clot covering the wound which constitutes a temporary matrix enabling the migration of inflammatory cells and protection against pathogens and fluid loss.^2^ Neutrophils are attracted to the wound within 24–36 h after injury by various factors secreted from the fibrin clot and damaged tissue, including transforming growth factor beta (TGF‐β), platelet‐derived growth factor (PDGF), epidermal growth factor (EGF), fibroblast growth factor (FGF), and interleukin (IL)‐8. Neutrophils secrete proteases, phagocyte bacteria present in the wound and degrade necrotic tissue.^3^, ^4^, ^5^ They are followed by monocytes, which differentiate into macrophages able to phagocytose cell debris and dead neutrophils.^6^ In the late inflammatory phase, macrophages secrete growth factors (TGF‐β, EGF, PDGF, FGF) and the proinflammatory cytokines IL‐1 and IL‐6, thus activating keratinocytes, fibroblasts and endothelial cells.^7^, ^8^ The second, proliferative phase, which begins 3–4 days after injury and lasts from 2 to 4 weeks, is stimulated by factors secreted in inflammatory phase. In the proliferative phase, angiogenesis and epithelialization occur and ECM and granulation tissue are formed.^9^ Angiogenesis, which is essential for the formation of granulation tissue, is induced by growth factors: vascular endothelial growth factor A (VEGF‐A), FGF‐2, PDGF, and TGF‐β.^10^ Collagen secreted by fibroblasts gradually replaces the fibrin matrix. Fibroblasts also differentiate into myofibroblasts expressing α‐smooth muscle actin, which enables wound contraction.^11^ Remodeling of the wound and surrounding tissues by fibroblasts, which is the final stage of wound healing, begins approximately 3 weeks after injury and can last up to 2 years.^12^ During remodeling, all processes activated in the earlier phases are terminated, and myofibroblasts, macrophages and endothelial cells undergo apoptosis.^13^ Collagen III is converted to collagen I by metalloproteinases, which, together with collagen rearrangement into an organized structure, leads to strengthening of the wound.^14^ Typically, wounds reach approximately 20% strength of healthy skin after 3 weeks, and 80% after 12 months.^15^

Many methods are used in the treatment of nonhealing wounds, including different kinds of dressings (e.g., films, hydrocolloids, foams, hydrogels, alginates, hydrofibers), tissue‐engineered skin substitutes, growth factors, negative pressure therapy, and hyperbaric oxygen.^16^ However they are not fully efficient. In recent years, much attention has been paid to the use of cell therapies in the treatment of wounds. For example, epidermal stem cells were first used in wound treatment in 1981 and are now applied to promote healing of burns and chronic wounds.^17^, ^18^ Autologous genetically modified cultured epidermal stem cells were also successfully used in a clinical trial for junctional epidermolysis bullosa.^19^, ^20^ Recently, therapies with mesenchymal stromal cells (MSCs), especially adipose‐derived MSCs (AD‐MSCs), have been of great interest around the world.^21^ For many years, adipose tissue has been considered medical waste but is in fact a great source of stem cells. AD‐MSCs are easy to obtain and have similar properties to bone marrow‐derived MSCs (BM‐MSCs).^22^ Adipose tissue is a more effective source of stem cells, which can be extracted in large amounts (500‐fold greater than BM‐MSCs when counted per unit volume of fat) without ethical concerns. Additionally, AD‐MSCs show higher proliferative capacity, longer life‐span and shorter doubling time than BM‐MSCs.^23^ Stem cells have great potential for chronic wound healing due to increased cell migration, high proliferative potential and release of cytokines and biological factors that regulate angiogenesis, induce repair processes, and inhibit inflammatory and immune responses.^24^, ^25^

There is some inconsistency regarding the nomenclature of MSCs and mesenchymal stem cells. According to the International Society for Cellular Therapy (ISCT) the term “mesenchymal stromal cells” refers to the plastic‐adherent fraction of cells showing immunomodulatory, secretory and homing properties, while “mesenchymal stem cells” refer to the population expressing progenitor properties such as self‐renewal and differentiation potential to multiple cell linages.^26^ The abbreviation “MSC” should be used with additional information of the tissue source origin of the cells, for example, AD‐MSCs (adipose tissue‐derived MSCs) and a functional definition should be provided to clarify whether it refers to stem or stromal cells (i.e., stemness confirmation with in vivo and in vitro tests).^27^ Additionally, because MSCs act therapeutically by homing in on the injury site and secreting immunomodulatory and regenerative factors, which makes them therapeutic drugs, *Caplan* ^28^ suggested naming them “medicinal signaling cells.”

In this review, we summarized current knowledge about chronic wound treatment with the use of AD‐MSCs with a particular focus on wound healing complications in diabetic and oncological patients. Diabetes and cancer are civilization diseases and the number of patients suffering from them is constantly growing. Wound healing problems are a common complication of diabetes and oncological treatment. Intensive research is underway all over the world on new drug compounds and methods of supporting wound healing in cancer patients and patients with diabetes. The review summarizes not only the biological characteristic of AD‐MSCs but also the technical aspects of their isolation, cell culture and transplantation to nonhealing wounds. A comparison of AD‐MSCs from different sources (subcutaneous and visceral adipose tissues [SAT and VAT]) was also made. Additionally, factors that can affect AD‐MSCs as well as ways to enhance their therapeutic potential were described.

## 2 CHRONIC WOUND CHARACTERISTICS AND CLINICAL NEED

**Figure 2:**
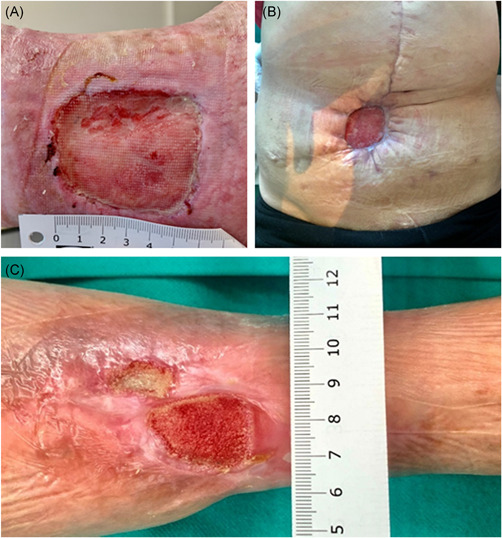
Chronic wounds of different etiology. (A) Posttraumatic chronic wound of lateral ankle, diabetic foot syndrome; (B) Postoperative wound of abdomen, operation of hernia in postoperative scar complicated by infection and necrosis of the abdominal wall, (C) Posttraumatic chronic wound of shank, ischemic wound [Color figure can be viewed at wileyonlinelibrary.com]

Chronic wounds are wounds that do not heal through normal wound healing phases in an orderly and timely manner for at least 1 month.^29^ Medical conditions, for example, diabetes, autoimmune diseases, vascular pathologies, obesity, neuropathy or infections, as well as therapeutics, such as cancer chemotherapeutic agents, radiation therapy, nonsteroidal antiinflammatory agents or glucocorticoids, can affect the wound healing process and lead to the formation of nonhealing or chronic wounds. Patient age, nutrition, smoking status and alcohol consumption are also important extrinsic factors.^30^ A distinction is made between wounds of various etiologies: venous and ischemic ulcers, diabetic foot syndromes, posttraumatic and postoperative wounds, pressure sores and burn wounds (Figure 2.).^31^, ^32^, ^33^, ^34^

Chronic wounds constitute not only a medical issue but also a large economic issue. In developed countries, 1%–2% of the general population has chronic nonhealing wounds.^35^ With an aging society and a growing population of patients suffering from diabetes, obesity and vascular diseases, these numbers are expected to rapidly increase.^36^ The quality of life of patients with chronic wounds is significantly impaired and they show high morbidity and mortality rates.^37^ In addition, treating these wounds is a serious economic burden, estimated to comprise approximately 1%–3% of medical budgets in developed countries.^38^

Chronic wounds of different etiologies possess several common features, such as elevated levels of proinflammatory cytokines, proteases, reactive oxygen species (ROS) or senescent cells, dysfunction or deficiency of stem cells, decreased levels of growth factors, abnormalities in ECM functions and weak blood supply.^39^, ^40^ Their healing process is mainly arrested in the inflammatory phase, and antimicrobial and phagocytic activity of the immune cells appears to be lower in chronic wounds than in acute wounds, which likely leads to the accumulation of necrotic tissue on the wound edges.^41^ The level of growth factors essential for proper wound healing can also be important in the formation of chronic wounds. For example, bFGF, PDGF, TGF‐β, and EGF levels are reduced in chronic pressure ulcers compared to acute wounds.^42^ In addition, chronic wounds are often gradually colonized by various bacteria, for example, *Staphylococcus aureus*, *Enterococcus faecalis*, and *Pseudomonas aeruginosa*, all of which form a biofilm enabling them to become not only resistant to antibiotics, other antimicrobial agents and the body's defense mechanisms but also more susceptible to other bacterial and fungal infections.^43^, ^44^, ^45^ The presence of bacteria and their toxins causes excessive inflammatory reactions and tissue damage and results in intensified local pain.^46^ In addition, immune cells and bacteria produce proteases that degrade ECM and growth factors in the wound.^47^

### 2.1 Wound healing in diabetes

Diabetes mellitus is a chronic metabolic disease characterized by hyperglycemia. According to the World Health Organization, 422 million adults suffered from diabetes in 2014, and 1.5 million people died of diabetes‐related complications in 2012.^48^ It is estimated that the population of diabetic individuals will grow to 592 million by 2035.^49^ In 2017, the cost of diabetes in the United States was 237 billion dollars, one‐third was allocated to the treatment of diabetic foot ulcers (DFUs).^50^ These wounds are one of the most common and serious complications of diabetes and a major cause of morbidity and mortality in individuals with diabetes. Ischemia, neuropathy and infection, often occurring together, constitute the etiological triad, which leads to complications of DFUs.^51^ Approximately 15% of people suffering from diabetes have diabetic ulcer during their lifetime, and 85% of amputations in diabetic patients are caused by foot ulceration, which further deteriorates to severe infection. Besides, 50%–70% of all lower limb amputations performed are the result of diabetes.^52^, ^53^

Hyperglycemia can affect wound healing through different mechanisms. Tissue loss may be aggravated by a neuropathic lack of sensation, and wound healing may also be delayed by dysfunctional epithelialization caused by impaired cell proliferation and resistance to growth factors.^54^ Impairment in many key processes for proper wound healing including the production of growth factors, the proliferation and migration of keratinocytes and fibroblasts, the angiogenic response, collagen accumulation or the balance between ECM component accumulation and remodeling, plays main roles in the pathophysiology of DFUs (Figure 1).^55^ In diabetic wounds, macrophage transition from a proinflammatory to an antiinflammatory state does not occur, and these cells remain mainly in a proinflammatory state.^56^ Prolonged inflammation is also caused by IL‐1β and tumor necrosis factor α (TNFα), whose levels are increased during the inflammatory phase and remain elevated in wounds for a longer time. The stability and activity of hypoxia‐inducible factor 1 affected by hyperglycemia results in the suppression of its target genes, for example, VEGF, and impaired in endothelial progenitor cell recruitment caused by decreased production of nitric oxide (NO) contributes to reduced angiogenesis.^57^, ^58^, ^59^ In addition, keratinocytes from the margins of diabetic ulcers are activated and highly proliferative (increased expression of Ki67) and do not express the differentiation markers keratins 2 and 10 (K2, K10). They also do not migrate and show reduced expression of the precursor of the α3 chain of laminin 5 (LM‐3A32), which is a molecule present in migrating epithelial cells.^60^ In vitro cell cultures of fibroblasts isolated from DFUs showed reduced motility, altered secretion of cytokines (lower levels of C‐X‐C motif ligand 1, IL‐6, IL‐8, IL‐23, monocyte chemoattractant protein 1 (MCP‐1), and stromal cell‐derived factor 1) and a drop in the maximum mitogenic response to growth factors compared to those of cells from nondiabetic patients.^61^, ^62^ Additionally, in diabetic wounds, reduced levels of many growth factors including PDGF, KGF, VEGF, insulin‐like growth factors 1 and 2 (IGF‐1, IGF‐2), nerve growth factor, TGF‐β1 and KGF, were observed, which may contribute to the delayed healing of these wounds.^63^ The results from a prospective cohort study on diabetic patients showed that serum levels of TNF‐α, MCP‐1, matrix metalloproteinase 9 (MMP‐9) and FGF‐2 were higher in patients with nonhealing ulcers than in those whose ulcers had healed. Moreover, increased immune cell infiltration and expression of MMP‐9 and protein tyrosine phosphatase 1B (PTP1B) were observed in skin biopsies of diabetic patients. These factors are associated with, for example, increased inflammation. MMP‐9 is involved in the degradation of proteins and growth factors, while PTP1B takes part in negative regulation of insulin, leptin, and growth factors signaling (e.g., PDGF, VEGF, and EGF). Increased expression of extracellular MMP‐9 and intracellular PTP1B may not only lead to local inactivation and resistance to the activity of growth factors but also, in a way similar to elevated levels of insulin in insulin resistance, to an increase in circulating growth factors levels.^64^ Additionally, impairment in the regulation of ECM was confirmed in diabetes. For example, reduced levels of collagen and elastin were found in biopsies from DFU edges. This changes probably arose as a result of persistent inflammation and fibroblast senescence as well as poor vascularization of the wound.^65^ Hyperglycemic conditions may also directly contribute to increased production of MMPs and a reduction of tissue inhibitors of metalloproteinases (TIMPs), which results in disruption of the structures essential for proper wound healing.^66^, ^67^

### 2.2 Wound healing in oncological patients

**Figure 3:**
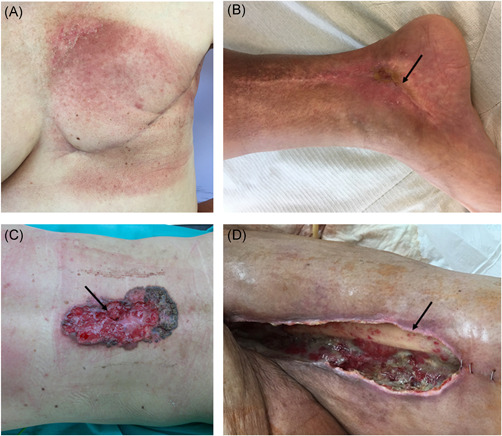
Skin complications in oncological patients. (A) 57‐year‐old patient after a radical mastectomy and postoperative radiotherapy due to breast cancer. The figure shows skin discoloration in the irradiated area, that is, at the breast and armpit. In the postoperative course, features of marginal necrosis of the wound treated in an outpatient setting were observed; (B) 35‐year‐old patient after a radical surgery of soft tissues sarcoma of a right lower leg above the ankle and a postoperative radiotherapy. Due to ischemia, the wound in the lower limb, in the area from the back and below the ankle, was accompanied by prolonged healing. The figure shows the place of impaired healing visible as a depression with a fragment of an atrophic wound (arrow); (C) 47‐year‐old patient with the ulceration of the back (dimensions:17 ×10 cm) resulting from skin cancer before the radical procedure. This type of neoplastic ulcer is associated with infection and necrosis. During radical surgery, the most important element is to protect the operated site from infection of the postoperative wound. The arrow marks the site of ulceration as a place with increased risk of postoperative wound infection; (D) Tissue loss of the left tight in a 63‐year‐old patient after a radical surgery of soft tissues sarcoma and postoperative radiotherapy. A fragment of the thigh bone with a defect in the thigh muscles is visible. It is the most difficult variant to heal, due to the extensive tissue loss and the condition after undergoing oncological treatment. The arrow marks the exposed femur [Color figure can be viewed at wileyonlinelibrary.com]

Cancer is one of the greatest challenges of modern medicine and the numbers of newly diagnosed cases and cancer‐related deaths are rapidly growing worldwide. It is estimated that 18.1 million new cases of cancer were diagnosed and that 9.6 million patients died because of cancer in 2018.^68^ In the treatment of cancer, chemotherapy and/or radiotherapy can be given to patients preoperatively (neoadjuvant therapy) or after surgical resection of the tumor (adjuvant therapy). Despite having many advantages, such as increased 5‐year survival rates and decreased numbers of local recurrence, neoadjuvant treatment may also cause postoperative complications in wound healing, resulting in reduced blood supply to the wound and regenerative potential as well as a higher incidence of infections. Usually, surgery is postponed to 4–6 weeks after neoadjuvant treatment, but it does not fully prevent the risk of such complications.^69^, ^70^ In oncological patients, skin manifestations, wound healing complications and tissue loss may either be caused by the tumor itself or result from the method of treatment used (Figure 3). A common problem is also infections at the surgery site, for example, after breast surgery, which can also delay healing.^71^

Chemotherapeutic drugs may interrupt processes responsible for proper wound repair by inhibiting cell division; metabolism and angiogenesis; the synthesis of DNA, RNA, and proteins; cell migration into the wound and the formation of wound matrix. In addition, they impair fibroblast growth, and inhibit collagen production and wound contraction.^72^ These drugs are designed to target rapidly dividing cells, so macrophages and fibroblasts are as susceptible to their activity as cancer cells.^73^ In addition, chemotherapy weakens patients' immune system which may interfere with the inflammatory phase of wound healing and increase the possibility of wound infections.^74^, ^75^ Targeted therapeutics such as epidermal growth factor receptor (EGFR) or VEGF inhibitors should be less toxic to normal cells, but their therapeutic targets involved in cancer growth also participate in wound healing, so the use of these therapeutics is associated with adverse reactions, including skin toxicity.^76^ Blocking EGFR signaling in healthy keratinocytes results in inhibition of their growth, proliferation, and migration.^77^ The use of bevacizumab, a monoclonal anti‐VEGF antibody, to block angiogenesis, may cause wound dehiscence, bleeding, and infections resulting from the limited delivery of cells, nutrients and oxygen. To avoid wound healing complications, it is recommended to perform surgery 60 days or 6–8 weeks after treatment with this drug and not to take bevacizumab at least 28 days after surgery.^78^, ^79^, ^80^

The side effects of radiotherapy can be categorized as acute (hours to weeks after exposure) and late (months to years after exposure). Acute injury includes hyperpigmentation and early ulceration, while late effects present as tissue fibrosis, necrosis, atrophy, vessel damage, and chronic ulcers.^81^ Skin damage is one of the most frequent acute side effects of radiotherapy, with 90% of patients who undergo radiotherapy developing skin reactions.^82^ The cytotoxic effect of radiation is a result of direct DNA ionization and interaction of produced ROS with DNA. Base alterations, the formation of dimers and DNA double‐strand breaks result in damage to basal keratinocytes and a reduction in the self‐renewing properties of the epidermis. Overexpression of proinflammatory factors, including TNF‐α, interferon gamma (IFN‐γ), IL‐1, IL‐3, IL‐5, IL‐6, IL‐8, and adhesion molecules, including intercellular adhesion molecule 1, vascular cell adhesion molecule, and E‐selectin sustain the inflammatory phase. Wound strength is decreased by the prevention TNF‐α and IFN‐γ‐mediated collagen deposition, the production of highly disorganized collagen and alterations in the production of ECM proteins resulting from changes in fibroblasts.^83^, ^84^, ^85^, ^86^ Moreover, elevated levels of TGF‐β in the serum of irradiated patients correlated with a higher risk of radiation‐induced fibrosis.^87^ Low levels of angiogenic factors (FGF, HGH, and VEGF) in irradiated skin may be responsible for inappropriate vascularization, disrupted angiogenesis and reduced blood supply.^88^ Persistent high concentrations of MMP‐2 and MMP‐9 and an imbalance of the expression of TIMPs and decreased angiogenesis may be the reason why these wounds fail to heal.^89^

## 3 CELLS AND TISSUE SOURCES

**Figure 4:**
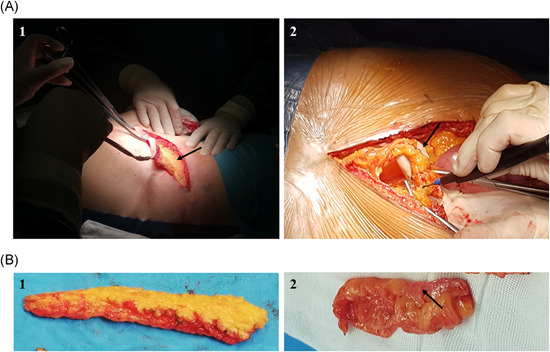
Fat collection through surgical resection. (A, 1) Collection of subcutaneous adipose tissue. Visualization of surgical wound during the process of fat tissue sample collection from subcutaneous tissue. Arrow points at the base of the wound which consists of fascial tissue of abdominal wall. (A, 2) Collection of visceral adipose tissue. Visualization of surgical wound after laparotomy procedure. The fat tissue sample is collected from the round ligament of the liver, arrow points at round ligament of the liver. (B, 1) Subcutaneous adipose tissue. (B, 2) Visceral adipose tissue. The photo shows intraperitoneal adipose tissue, the fragment is coated with the serosa (arrow) [Color figure can be viewed at wileyonlinelibrary.com]

Adipose tissue may be obtained in two ways: surgical resection of excess fat tissue (Figure 4) or liposuction of lipoaspirate. The techniques of fat harvesting are summarized in Table 1. Lipoaspirate from liposuction can be directly clinically used without previous AD‐MSCs in vitro culturing.^90^ The harvesting procedure uses a tumescent solution of the acidic pH including which includes Klein solution (see below), lidocaine or epinephrine which may induce a perioperative and postoperative burning sensation. The latest studies show that the presence of lidocaine decreases AD‐MSCs survival by an apoptosis increase. The neutralization of the tumescent solution by adding sodium bicarbonate can increase the survival of AD‐MSCs (by an increase in the number of viable cells and apoptosis decrease) and the stromal vascular fraction (SVF, up to 53%) and attenuates perioperative and postoperative pain.^91^ Avoiding in vitro manipulation of clinical material seems to be safer and bypasses some legal regulation.^92^

**Table 1:** Techniques of fat harvesting

Method of fat harvesting	Advantages	Disadvantages
Vacuum aspiration	Short time to harvest a larger volume of fat tissue Small scar	High negative pressure results in trauma to the adipocytes Necessity to have vacuum equipment
Syringe aspiration Coleman technique	Gentle negative pressure, which minimizes trauma to the adipocytes Small scar The possibility of taking a large amount of fat tissue in local anesthesia	Longer time to obtain same amount of fat tissue compared to the vacuum aspiration method
Surgical excision	Maintains the structure and viability of harvested fat tissue by avoiding damage to the adipocytes	Bigger scar General anesthesia must be used to excise more fat tissue Lower tissue plasticity

The type of harvesting procedure influences the yield, viability and proliferation of AD‐MSCs. There are few main isolation methods: mechanical‐assisted liposuction (MAL), power‐assisted liposuction (PAL), laser‐assisted liposuction (LAL), ultrasound‐assisted liposuction (UAL) and surgical resection. PAL has been identified as the best method because cells isolated using this technique show high proliferation potential and low senescence. A comparison between MAL and UAL‐derived AD‐MSCs did not indicate significant changes in the expression of MSC markers (CD13, CD29, CD73, CD90, CD105; only the level of CD166 was higher in UAL‐derived cells). Other comparisons (surgical resection, PAL, and LAL) confirmed that different harvesting methods do not change the expression of basic mesenchymal markers (such as CD90 and CD40).^93^, ^94^, ^95^ AD‐MSCs obtained from the abdomen through resection or liposuction yield more cells than either UAL or fat tissue collected from the hip/thigh district.^96^

### 3.1 Isolation of AD‐MSCs

**Figure 5:**
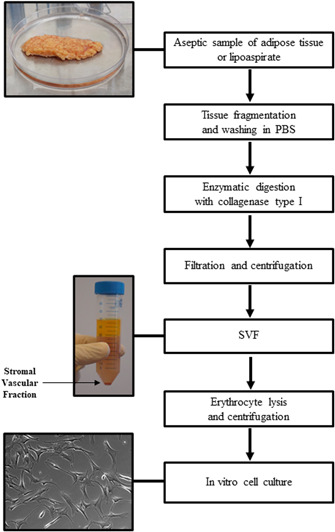
Isolation of AD‐MSCs by enzymatic method. AD‐MSC, adipose‐derived mesenchymal stromal cell [Color figure can be viewed at wileyonlinelibrary.com]

Cell isolation is a critical step in obtaining cells for experimental and therapeutic procedures. Enzymatic or nonenzymatic methods of AD‐MSCs isolation are described in the literature; the enzymatic method utilizing collagenase (Figure 5) is most frequently used. However, proteolytic enzymes, for example, trypsin‐EDTA, dispase or collagenase may negatively affect the viability and surface antigen expression on AD‐MSCs.^97^ Moreover, similar to fetal bovine serum (FBS) utilization, there are safety issues regarding the use of xenogeneic collagenase. To overcome this problem, nonenzymatic methods such as sonication, explant culture, and centrifugation were developed. However, none of the proposed nonenzymatic methods isolated the same number of cells as the enzymatic method.^98^ Comparison of the explant culture method and enzymatic method showed that the former had higher hematopoiesis potential, a lower percentage of CD34 expression and better quality, but required a large amount of lipoaspirate and resulted in lower overall yield of recovered cell.^99^ The enzymatic method elicited a significantly higher number of cells, higher colony‐forming efficiency (higher Nanog and Oct4 expression) and better differentiation capability.^100^ As we believe that the isolation efficacy is critical, we assume that enzymatic isolation is the best way of obtaining cells for clinical use. Therefore, it is important to develop protocols for AD‐MSCs isolation with clinical grade collagenase. Carvalho et al.^101^ showed that xeno‐free enzymatic products containing collagenases (e.g., Liberase TM) are as effective as research‐grade products in the isolation of AD‐MSCs. They did not notice statistically significant differences in cell properties (proliferation, differentiation, cell surface markers) among the cells isolated with different enzymatic products (CLS1 [Worthington], CLSAFA [Worthington], NB4 [Serva], Liberase [Roche]). Moreover, in 2013, a clinical trial using ex vivo expanded AD‐MSCs isolated with clinical collagenase NB 6 good manufacturing practice (GMP) grade (SERVA Electrophoresis GmbH) was performed to analyze the effect of fat grafts enrichment with AD‐MSCs on graft survival. The procedure showed good feasibility and safety.^102^

The efficiency, proliferation and pluripotency of AD‐MSCs depends on the donor site area. Analysis of cells from adipose tissue taken by liposuction from the abdomen, flanks, thighs, and medial knees showed that the abdominal region and inner thighs (expanding region during excess caloric intake) produce the highest yield of AD‐MSCs. The superficial abdominal depot is less sensitive to apoptosis and has higher differentiation potential compared to other subcutaneous depots.^103^, ^104^ AD‐MSCs can also be obtained from atypical locations, for example, buccal fat pads. Cells from this area express high levels of osteogenic markers; therefore, they seem to be more appropriate for treating bone defects.^105^ Comparison of AD‐MSCs lipoaspirates taken from the abdomen and hump showed differences: hump‐derived AD‐MSCs are smaller in size and narrower in overall appearance than are abdominal AD‐MSCs.^106^ Additionally, some differences have been observed between subcutaneous AD‐MSCs taken from the medial thigh or trochanteric area and those taken from the upper arm, which express higher levels of peroxisome proliferator‐activated receptor gamma (PPAR‐γ‐2).^107^

#### 3.1.1 SVF versus AD‐MSCs

The advantage of SVF over AD‐MSCs is its heterogeneous cellular composition (preadipocytes, fibroblasts, vascular smooth muscle, endothelial cells, macrophages, and lymphocytes), which is responsible for better therapeutic outcomes, comparable safety and less regulatory criteria. During culture expansion, AD‐MSCs can change surface marker expression and cells morphology. However, AD‐MSCs can be used in allogeneic and autologous treatments, while SVF can be used only in autologous treatment (Table 2).^108^, ^109^, ^110^ Allogeneic AD‐MSCs carry minimal rejection risk, and obtaining AD‐MSCs is less deleterious than obtaining other types of MSC.^111^ Furthermore, fluids secreted by acute and chronic wounds have an impact on AD‐MSCs and may regulate their regenerative potential.^112^

**Table 2:** Comparison of AD‐MSCs and SVF

	Treatment	Cell population	Cell types	Properties	Ex vivo exposure
AD‐MSCs	Allogenic and autologous	Homogenous	AD‐MSCs	Immunomodulatory, differentiative	High (weeks)
SVF	Only autologous	Heterogenous	AD‐MSCs, fibroblasts, vascular smooth muscle, endothelial cells, macrophages, lymphocytes	Immunomodulatory, differentiative, angiogenic	Low (hours)

Abbreviations: AD‐MSC, adipose‐derived mesenchymal stromal cell; SVF, stromal vascular fraction.

For clinical application, it is important to properly store the SVF and AD‐MSCs for later therapeutic use. Only an elongated period of cryopreservation at −70°C (>2 years) reduces the number of live cells and their viability.^113^ Compared to fresh lipoaspirate‐derived SVF, SVF from cryopreserved lipoaspirate has reduced cell viability and a lower colony‐forming‐unit percentage (16‐fold).^114^ It was shown that cryopreservation media containing human serum (HS) albumin, HS, or knockout serum replacement has no influence on AD‐MSCs conditions (gene expression, immunophenotype, and differentiation ability) for up to 3–4 freeze‐thaw cycles. However, their proliferation was significantly reduced, and it was suggested to perform no more than two freeze‐thaw cycles on cells for clinical application.^115^

## 4 CHARACTERIZATION OF AD‐MSCs

The characterization of AD‐MSCs is critical before their application in not only laboratory research but also the clinic. Culture conditions can affect the properties of these cells and thus their therapeutic potential.

### 4.1 Markers and differentiation potential of AD‐MSCs

Since the discovery and isolation of MSCs populations from adipose tissue, much effort has been paid to their characterization, especially in the identification of their surface markers. According to the statement from the ISCT, MSCs are defined by minimal criteria, including adherence to plastic, specific expression pattern of markers (positive [>95%]: CD105, CD73 and CD90, negative [<2% positive]: CD45, CD34, CD14, or CD11b, CD79a or CD19 and HLA Class II) and multipotent differentiation potential (differentiation into adipocytes, osteoblasts and chondroblasts under standard differentiation conditions).^116^ Later, the ISCT with the International Federation for Adipose Therapeutics and Science (IFATS) published guidelines for the characterization of AD‐MSCs. According to these guidelines, cells in SVF should be identified by the following markers: CD45^−^CD235a^‐^CD31^‐^CD34^+^ and additional expression of CD13, CD73, CD90, and CD105. In turn, AD‐MSCs in cell culture should have expression pattern akin to that of other MSCs: positive for CD90, CD73, CD105, and CD44 and negative for CD45 and CD31 (Table 3). Expression of CD36 and a lack of CD106 expression can be used to distinguish AD‐MSCs and BM‐MSCs.

**Table 3:** Immunophenotype of SVF and AD‐MSCs according to IFATS and ISCT^117^

	SVF		AD‐MSCs	
Positive markers	Primary stable	CD13, CD29, CD44, CD73, CD90 (>40%), CD34 (>20%)	Primary stable	CD13, CD29, CD44, CD73, CD90, CD105 (>80%)
			Primary unstable	CD34 (present at variable levels)
			Other markers	CD10, CD26, CD36, CD49d, CD49e
Negative markers	Primary negative	CD31 (<20%), CD45 (<50%)	Primary negative	CD31, CD45, CD235a (<2%)
			Other markers	CD3, CD11b, CD49f, CD106, PODXL

Abbreviations: AD‐MSC, adipose‐derived mesenchymal stromal cell; SVF, stromal vascular fraction; IFATS, International Federation for Adipose Therapeutics and Science; ISCT, International Society for Cellular Therapy.

**Figure 6:**
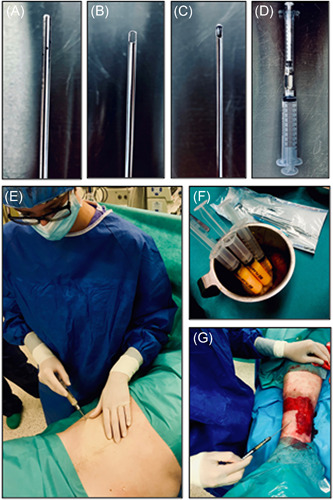
Fat grafting by Coleman technique. (A) Infiltration cannula; (B) Harvesting canula; (C) Fat transfer canula; (D) Syringe for fat harvesting connected with insulin syringe for fat transfer with a Luer‐ Lock connector; (E) Harvesting fat tissue—Coleman technique; (F) Lipoaspirate; (G) Autologous fat grafting into the chronic wound (our preliminary trials in patients, approved by the Independent Bioethics Commission for Research of the Medical University of Gdansk—permission number NKBBN/707/2018‐2019) [Color figure can be viewed at wileyonlinelibrary.com]

In addition, the differentiation potential of AD‐MSCs into adipocytes, osteocytes and chondrocytes should be qualitatively analyzed by histological staining, and additional quantitative analyses with biochemical methods (Western blot, enzyme‐linked immunosorbent assay) or RT‐PCR should be considered (Figure 6).^117^

There is some controversy regarding the CD34 marker, whose expression on AD‐MSCs is unstable. MSCs should be negative for these markers, however, the results of various studies indicate their differential expression.^118^ In the SVF, a large percentage of cells (up to 85%) were shown to be CD34^+^.^119^, ^120^ Some authors confirmed that cultured AD‐MSCs do not express CD34,^121^, ^122^, ^123^ while others reported some fractions of AD‐MSCs to be CD34^+^.^124^, ^125^ Comparison of sorted fractions of early passages of cultured human AD‐MSCs showed that the CD34^+^ cells had greater proliferative potential and colony‐forming ability whereas CD34^−^ cells were characterized by greater differentiation potential into osteocytes and adipocytes.^126^ In our studies, in flow cytometric and quantitative polymerase chain reaction (qPCR) analyses, we observed little or no CD34 expression in AD‐MSCs cultured in vitro up to the sixth passage.^127^ CD34 expression was confirmed to be affected by culture conditions (e.g., plating density and culture medium) and decreased during cell culture.^128^, ^129^

### 4.2 Genetic stability of AD‐MSCs and effect of FBS deprivation on AD‐MSCs

The genetic stability of AD‐MSCs during cell culture should be addressed due to their clinical application. In clinical trials, cells up to the second passage are usually used, but sometimes longer in vitro culture is required. During cell culture, the proliferative and differentiation potential of cells can diminish. Reduced DNA synthesis and DNA repair efficiency may lead to the accumulation of DNA damage, genetic instability, cell senescence, and functional changes and consequently affect the therapeutic efficacy and patient safety.^130^ Rubio et al.^131^ showed that AD‐MSCs can undergo spontaneous transformation in in vitro cell culture. However, the culture was continually passaged for 4–5 months. Our studies showed that in a long‐term (up to sixth passage) in vitro cell culture, AD‐MSCs from different donors (plastic surgery and oncological patients) maintain their differentiation potential (i.e., ability to differentiate into adipocytes, osteocytes and chondrocytes) and retain their phenotype based on the expression of key surface markers at the transcript (qPCR) and protein level (flow cytometry). These results confirm AD‐MSCs stability and safety during long‐term culture.^127^ AD‐MSCs stability was also demonstrated by other research teams. Neri et al.^132^ observed an expected slowdown of the proliferation rate during long‐term culture but no instances of genetic changes (alterations in chromosome or short repeated sequences), replicative senescence (telomere attrition, expression of significant amounts of active telomerase) or anchorage‐independent growth ability, which indicates the therapeutic safety of AD‐MSCs. It was also indicated that AD‐MSCs do not show signs of senescence up to the seventh passage regardless of culture conditions (oxygen tension and medium supplementation with FBS or human platelet lysate).^133^

Another important issue regarding AD‐MSCs application is the evaluation of the effect of FBS deprivation on these cells. Traditionally, cells are cultured in medium supplemented with FBS. However, it is recommended to avoid FBS during the culture of cells for preclinical and therapeutic applications. To be considered safe, cells have to be cultured according to GMP standards, so animal‐related products need to be eliminated. This is due to the risk of cell product contamination with infectious agents. In addition, FBS contains various growth factors, hormones, nutrients and other components, and its composition varies significantly between batches. FBS use may lead to unspecific activation of cell differentiation and proliferation as well as immune responses.^134^, ^135^, ^136^ Accordingly, FBS is removed from cell culture before clinical application and during testing of drug candidates.^137^, ^138^ The available results show, that AD‐MSCs maintain their stem cell characteristics in serum‐deprived medium; they survive, proliferate and are able to differentiate.^139^, ^140^ Our research on AD‐MSCs after the second passage, which were cultured in the absence of FBS for 48 h, comprised whole transcriptome sequencing followed by gene expression analysis and showed that FBS‐deprived AD‐MSCs, at the transcription level, show lower metabolic and proliferative activity but retain the expression of key surface markers. Additionally, we did not find evidence of apoptosis and necrosis. These observations suggest that FBS deprivation does not induce changes that could preclude the clinical application AD‐MSCs.^127^

To overcome the problem of FBS use in cell culture, media containing a combination of recombinant growth factors are used. For example, commercially available chemically modified STK2 medium (DS Pharma Biomedical), which contains FGF2, PDGF, EGF, insulin, lipids, nutrients and minerals, is provided for AD‐MSCs in vitro cell expansion.^141^ AD‐MSCs cultured in STK2 medium showed a higher proliferation rate, increased expression of MSC surface markers and reduced senescence than AD‐MSCs grown in DMEM supplemented with FBS.^142^ However, serum‐free media are mainly applied in laboratory research and not in clinical trials, and the safety of serum‐free media in the clinic needs to be evaluated.^143^ Other supplements, such as pooled human AB serum, platelet lysates, cord blood serum or thrombin‐activated platelet rich plasma (tPRP) were tested as alternatives for FBS in AD‐MSCs cell culture. Dessels et al.^136^ proved that compared to cells cultured in medium with FBS, AD‐MSCs expanded in pooled human platelet lysates (pHPLs) were characterized by higher expression levels of genes involved in the cell cycle, proliferation and division. Additionally, pHPL supplementation did not affect the expression levels of genes responsible for the differentiation of specific developmental processes. Similar findings were also presented in other studies.^144^, ^145^ In addition, Kocaoemer et al.^146^ reported that AD‐MSCs culture in medium supplemented with pooled human AB serum or tPRP increased their proliferative capacity without compromising their immunophenotype and differentiation potential. However, cells cultured in tPRP‐supplemented medium showed decreased adhesion. Additional whole genome gene analysis revealed significantly higher expression levels of 90 genes in AD‐MSCs cultured in FBS‐supplemented medium. Moreover, cells grown in medium containing human supplements showed lower expression of adhesion‐ and ECM‐associated molecules.^147^ In turn, Lindroos et al.^148^ indicated that AD‐MSCs cultured in allogeneic HS (allo‐HS) and FBS show comparable proliferative potential and phenotypes, but there were differences in the gene expression patterns between those culture methods (overexpression of cell cycle pathway genes in allo‐HS culture and BMP receptor‐mediated signaling in the TGF‐β pathway in FBS culture). Culture conditions can affect AD‐MSCs properties, therefore, there is still a need to look for appropriate xeno‐free culture media that will provide the right number of cells for clinical application without affecting their therapeutic potential.

### 4.3 SAT versus VAT

Subcutaneous adipose tissue (SAT) and VAT have different characteristics and functional roles in metabolic regulation, for example, VAT has a higher inflammatory response than SAT.^149^ Differences between these kinds of fat tissue are probably based on other developmental origins: SAT originates from somatic lateral mesoderm while VAT originates from splanchnic lateral mesoderm.^150^ AD‐MSCs obtained from visceral (V‐AD‐MSCs) or subcutaneous fat tissue (S‐AD‐MSCs) exhibit no difference in reconstructive potential, but collecting VAT is more invasive for patients (Table 4).^103^ Single‐cell RNA‐seq analysis showed different transcriptomic features between S‐AD‐MSCs and V‐AD‐MSCs (S‐AD‐MSCs have larger DPP4‐positive subpopulation). AD‐MSCs from both tissues show diverse fibrosis, adipogenesis, vascularization and inflammation outcomes and have different, heterogeneous gene expression profiles. V‐AD‐MSCs are more adipogenic and proinflammatory while, S‐AD‐MSCs are more like progenitor cells with antiinflammatory properties.^151^, ^152^

**Table 4:** Comparison of S‐AD‐MSCs and V‐AD‐MSCs

	Kind of white fat tissue	Developmental origin	Inflammatory cytokines	Type of cell	Differentiation into adipocytes/osteocytes
S‐AD‐MSCs	Subcutaneous	Somatic lateral mesoderm	Antiinflammatory	Progenitor	Lower
V‐AD‐MSCs	Visceral	Splanchnic lateral mesoderm	Proinflammatory	Adipogenic	Higher

Abbreviations: AD‐MSC, adipose‐derived mesenchymal stromal cell; V‐AD‐MSC, AD‐MSC obtained from visceral adipose tissue; S‐AD‐MSC, AD‐MSC obtained from subcutaneous adipose tissue.

S‐AD‐MSCs and V‐AD‐MSCs share similar surface markers and cell viability but differ in functions (depending on the surrounding microenvironment), motility, focal adhesion dynamics, secretion of inflammatory cytokines, and expression of stemness‐related genes.^153^ Compared to V‐AD‐MSCs, S‐AD‐MSCs have a higher potential to differentiate into adipogenic and osteogenic cells but V‐AD‐MSCs secrete higher levels of inflammatory cytokines (IL‐6, IL‐8) and TNF‐α.^154^

Raajendiran et al.^155^ identified three distinct human adipocyte progenitor cell (APC) subtypes according to the expression of CD34 (CD34^−^, CD34^low^, CD34^high^). Performed analysis showed that AD‐MSCs in visceral fat tissue depot have an equal number of CD34^−^ and CD34^hi^, while in abdominal, subcutaneous depot AD‐MSCs are highly enriched with CD34^−^, followed by CD34^hi^ and CD34^lo^. S‐AD‐MSCs are specifically characterized by CD10 expression, while V‐AD‐MSCs predominantly by CD200.^156^ The adipogenic capacity in cells is correlated with CD10 positivity and CD200 negativity, which agree with better differentiation of S‐AD‐MSCs than V‐AD‐MSCs in response to in vitro adipogenic stimuli.^156^, ^157^ V‐AD‐MSCs proliferate slower and need stronger stimulation to differentiate.^157^ Its weaker capacity to differentiate into new adipocytes could partly explain the hypertrophy of existing adipocytes in VAT as a response to fat accumulation in obesity.^158^ Additionally, V‐AD‐MSCs show a reduction of CD90, which could mediate metabolic disorder. S‐AD‐MSCs express a high level of CD90 and show an increase in proliferation, mitotic clonal expansion, and adipogenic differentiation, together with AKT activation and G1‐S phase transition, which may contribute to metabolic homeostasis via preventing adipocyte hypertrophy in SAT.^159^

Wada et al.^160^ indicate differences in the angiogenesis and inflammatory cytokines release pattern between S‐AD‐MSCs and V‐AD‐MSCs. The secretion of cytokines, for example, CHI3LI; IL‐1β; EGF; MCP‐1; CST3; IL‐6; IL‐8; PTX3; TGF‐β; PLAUR, and TNF‐α was smaller in the supernatants of the S‐AD‐MSCs. Researchers also show that the S‐AD‐MSCs proliferate 1.75 times faster than the V‐AD‐MSCs. The insulin‐producing cells (IPCs) for glucose stimulation, generated from the V‐AD‐MSCs, and S‐AD‐MSCs, showed that insulin secretion capacity was higher in the subcutaneous IPCs than the visceral IPCs.

Both S‐AD‐MSCs and V‐AD‐MSCs from obese and Type‐2‐diabetic patients show higher migration, invasion, and phagocytosis capacity than those from lean subjects. The weight loss in visceral and subcutaneous fat depots is able to at least partially restore their metabolic homeostasis.^158^

The above differences should be considered in regenerative therapies.

### 4.4 BAT versus WAT AD‐MSCs

Brown adipose tissue (BAT) which develops early in life is most prominent in human newborns and until recently, it was widely believed that BAT disappears by adulthood.^161^ In adults human, it is concentrated in the supraclavicular, neck, axillar, and paravertebral regions and it is inversely correlated with body mass index (BMI).^162^, ^163^, ^164^

Brown adipocytes are characterized by the presence of a large number of mitochondria, multilocular lipid droplets, sympathetic innervations, and the expression of uncoupling protein‐1 (UCP1) which allows generating heat with little ATP production.^161^, ^165^ Brown adipose develops from paraxial mesoderm (dermomyotome) similar to muscles and dorsal dermis.^166^

The Ebf2 (early B‐cell factor 2) transcription factor is a highly specific marker expressed in both BAT and beige/brite precursors, used for BAT identity and efficient brite/beige cell formation.^164^, ^166^ WAT can change to BAT in response to cold exposure or other stimuli (i.e., β‐adrenergic) that increase sympathetic tone.^161^ Cells from white fat tissue that show Ebf2 expression differentiate into brown‐like (or beige) adipocytes, what's more loss of Ebf2 in brown preadipose cells reduce the expression levels of brown preadipose‐signature genes. These results indicate that Ebf2 specifically marks and regulates the molecular profile of brown preadipose cells.^166^ Human abdominal subcutaneous white‐fat preadipocytes have greater brown‐adipocyte lineage commitment potential following BMP‐7 induction than preadipocytes isolated from visceral white fat.^162^ The mesenchymal progenitors that give rise to beige adipocytes express a unique set of cytokines and transcriptional regulators involved in immune cell modulation of adipose tissue browning. An iron accumulation and withstanding with oxidative stress suggest that beige/brite adipocytes are adapted to mitochondrial biogenesis and fatty acid oxidation upon thermogenic stimulation.^167^

WAT to BAT conversion is induced in response to β‐adrenergic receptor stimulation by β‐adrenergic agonist (e.g., norepinephrine,^168^ PPAR agonists,^169^ mirabegron,^170^ CL‐316,243,^171^ BRL 26830A^172^).

Multipotent metabolically active BAT‐derived stem cells were identified in adult humans mediastinum.^173^ BAT‐derived MSCs (BAT‐MSCs) differ from stem cells derived from WAT in the origin and lineage characteristics, particularly, WAT‐stem cells originate from Myf5 (myogenic regulatory factor) negative progenitors, whereas BAT‐MSCs express Myf5 and originate from myogenic lineage.^173^, ^174^ BAT‐MSCs are metabolically active, can be expanded in vitro, exhibit multilineage differentiation potential and can be functionally differentiated into metabolically active brown adipocytes. The BAT‐MSCs compared to the WAT stem cells have a unique gene expression profile, especially the higher expression of genes associated with BAT such as PPAR‐γ coactivator 1‐alpha (PGC‐1a), PR domain containing 16 (PRDM16), CAMP responsive element binding protein one (CREB1), and UCP1. Cells exhibit the capacity to undergo osteogenesis, chondrogenesis, and both brown and white adipogenesis.^173^

## 5 FACTORS THAT CAN AFFECT AD‐MSCs

Obesity, age and related chronic diseases can negatively affect AD‐MSCs. The properties of AD‐MSCs differ among fat depots and change with age.^175^ Cells from younger patients proliferate faster, are more successful in differentiating into mature adipocytes and have more lipolysis activity.^176^ Higher adipogenesis was shown in the middle‐aged group (40–45 years old vs. compared with 25–30 years old and 55–60 years old). The younger group demonstrated lower apoptosis susceptibility, the lowest of which was observed in the superficial abdominal depot. Cells isolated from elderly patients have lower function and adipogenic potential, while those isolated from younger individuals (25–30 years old) have a higher growth rate and paracrine activity. Additional comparisons showed that in the elderly patients, higher differentiation potential stays substantial only in the arm and subcutaneous thigh.^107^, ^177^

### 5.1 Obesity

AD‐MSCs from obese patients show reduced function and differentiation potential compared to those from lean controls.^178^ Louwen et al.^179^ showed that obesity has an unfavorable impact on AD‐MSCs, that is, defective functionalities and properties (differentiation, angiogenesis, motility, multipotent state, metabolism, and immunomodulation). Additionally, the undifferentiated multipotent state of AD‐MSCs is impaired in obese individuals. AD‐MSCs from obese individuals have upregulated adipogenic and inflammatory genes, enhanced epithelial‐mesenchymal transition, reduced expression of multipotency‐associated genes (e.g., OCT4, SAL4, SOX15, and KLF4), and decreased telomerase activity and telomere length (self‐renewal capacity). A higher BMI decreases AD‐MSCs osteogenesis potential and impairs angiogenic potential. Obesity also alters AD‐MSCs secretome profile due to its association with the proinflammatory environment, which can negatively impact on AD‐MSCs differentiation potential and regenerative capability.^180^, ^181^ Compared to obese AD‐MSCs, lean‐derived AD‐MSCs showed reduced immunosuppressive activities and weaker suppression of lymphocyte proliferation, which protects against obesity‐associated inflammation and insulin resistance.^182^

### 5.2 Cancer

AD‐MSCs and cancer cells show bidirectional effects. Tumor cells change the AD‐MSCs phenotype and function in a paracrine way. In coculture with lung cancer cells (H358), AD‐MSCs differentiate into myofibroblasts.^183^ Similar differentiation of AD‐MSCs was observed with cells cultured in presence of breast cancer exosomes, breast tumor‐derived factors and ovarian cancer exosomes.^184^ Wang et al.^185^ showed that AD‐MSCs adipogenesis and adipogenic‐specific genes are strongly inhibited by internalization of lung cancer‐derived exosomes. AD‐MSCs from patients with urological neoplasms show equivalent mesenchymal surface markers, exosome miRNA content, molecular karyotyping and similar growth kinetics to AD‐MSCs from healthy subjects, thus confirming the proper use of autologous stem cell transplantation in clinical treatment.^186^ In our experiments, we also did not observe differences in the differentiation potential and expression of key MSC markers at the transcriptome and protein levels in in vitro cultured AD‐MSCs from plastic surgery and oncological patients.^127^

Some in vivo and in vitro preclinical studies indicate AD‐MSCs as a factor that increases tumor growth and progression.^187^ The interaction between AD‐MSCs and the tumor microenvironment has been confirmed in patients with obesity, colorectal tumors, prostate tumors, melanoma and breast cancer, all of which show a higher quantity of circulating AD‐MSCs. Tumor stroma and inflammatory cells can release factors (e.g., SD‐1 and MCP‐1) that stimulate AD‐MSCs migration to the cancer microenvironment.^188^ AD‐MSCs can increase prooncogenic risk but do not differentiate or stimulate angiogenesis in cancer cells.^189^ It is suggested that AD‐MSCs promote tumor progression and invasiveness, but clinical trials have failed to demonstrate their prooncogenic potential.^163^ By contrast, some studies have shown that ASC exosomes exert anticancer immunomodulatory functions and decrease cancer growth, migration and colony formation.^190^

### 5.3 Chemotherapy/radiotherapy

It is important to evaluate the impact of oncological treatment on AD‐MSCs in the context of their potential autologous transplantation into the wounds in these patients. In mouse model, it was shown that whole‐body irradiation can damage adipose tissue and reduce the cell number and proliferative potential of AD‐MSCs.^191^ In vitro external radiation reduced the proliferation of AD‐MSCs, but the effect was smaller than that for normal human fibroblasts (NHFs). In the coculture of these cells, external radiation did not significantly reduce cell proliferation, which suggests that AD‐MSCs may protect NHFs and promote their growth.^192^ AD‐MSCs cocultured with NHF and microvascular endothelial cells (HDMECs) showed increased expression of cytokines and adhesion molecules (in NHFs and HDMECs) after radiation, which suggests that AD‐MSCs may have a stabilizing effect on irradiated wounds.^193^ The injection of AD‐MSCs is a promising therapeutic strategy in wound healing, especially after laryngectomy combined with radiotherapy.^194^ Fat transfer can lead to the healing of chronic ulcers resistant to other forms of postradiation treatment ^195^

Chemotherapeutic agents may also affect AD‐MSCs. Tamoxifen, a hormonal therapeutic widely used in breast cancer treatment, was shown to inhibit proliferation and induce apoptosis of AD‐MSCs in a time and dose‐dependent manner. Additionally, their ability to differentiate into adipocytes and osteocytes was impeded.^196^ On the other hand, AD‐MSCs are relatively resistant to commonly used chemotherapeutic agents (cisplatin, vincristine, and camptothecin) in vitro. After exposure to cisplatin and camptothecin at concentrations that reflect clinically relevant doses, AD‐MSCs maintained their stem characteristics (surface markers and osteogenic and adipogenic differentiation). They were also able to fully recover after treatment with high doses of cisplatin, vincristine, and camptothecin, which indicates that they are resistant to genotoxic damage in vitro.^197^

It is worth noting, that most of the research regarding the effect of chemotherapy or radiotherapy on AD‐MSCs was conducted in in vitro cell or in vivo animal models. This research is valuable but does not fully reflect the state of these cells in the human body. Thus, it is important to perform more extensive and detailed analyses of the properties of AD‐MSCs isolated from adipose tissues obtained from patients subjected to oncological treatment.

## 6 THE ROLE OF AD‐MSCs IN WOUND HEALING

AD‐MSCs play an essential role in wound healing, however, the mechanism of their action is still under investigation. Endogenous AD‐MSCs may be activated after wounding. During the proliferative phase mature adipocytes and their precursors, together with fibroblasts, populate the wound site.^198^ AD‐MSCs can also differentiate into fibroblasts, keratinocytes and endothelial cells and secrete various factors (e.g., cytokines and growth factors) that stimulate the proliferation and migration of these cells. In addition, through the secretion of growth factors (e.g., VEGF, bFGF, EGF, PDGF, hepatocyte growth factor, TGF‐α), cytokines (e.g., IL‐6, and IL‐8) and chemokines in a paracrine manner, AD‐MSCs can promote angiogenesis, the immune response, epithelial regeneration, and wound remodeling.^199^ They were also reported to exert antioxidant effects in wound healing.^200^

### 6.1 Fat grafting by the Coleman technique

Fat grafting was first described by Coleman as a cosmetic facial filler in the 1980s. In 1994, Coleman first introduced his technique of processing fat for structural fat grafting. This technique is called the Coleman technique (Figure 6) or structural fat grafting or “lipostructure” and uses syringes, cannulas, centrifuges and centrifugation protocols. Fat can be harvested under local or general anesthesia, depending on patient preference, pain tolerance and the volume of fat needed.^201^, ^202^, ^203^

The first step is to prepare fat tissue for harvesting and transplantation. For this purpose, fat tissue needs to be infiltrated through the miniature holes in skin, with a specialized cannula (Figure 6A) with a solution, known as Klein solution, which consists 0.5% lidocaine, 1:1000 epinephrine, sodium bicarbonate and Ringer's solution.^204^, ^205^

The next step is to uptake fat tissue by gentle manual suction of fat with 10 ml Luer‐Lock syringes and the specialized Coleman cannula (Figure 6B). The plunger of the syringe is gently pulled back to create light negative pressure to harvest the fat. This method produces a very low and constant vacuum that minimizes the destruction of adipocytes (Figure 6E).^206^

The lipoaspirate is processed for removal of the lipid and aqueous portions to isolate the adipose stroma for grafting. There are a few techniques for this isolation process, such as centrifugation, decantation, sedimentation, filtration, and mesh/gauze rolling; the Coleman protocol recommends centrifugation. Freshly harvested fat is centrifuged using appropriate gravitational force (3000*g* for 3 min) to separate the fat from unnecessary pollutants and nonviable components. Processed fat is transferred to 1 ml syringes and is ready for placement using the specialized cannulas (Figure 6D). There are several types of cannulas with different diameters, lengths and ends depending on the tissues into which the lipoaspirate is grafted.^207^, ^208^, ^209^

Coleman fat grafts have a greater number of viable adipocytes and sustain better cellular function than fat grafts harvested with other methods, and the Colman technique is currently the most common method of autologous fat transfer.^210^, ^211^ This procedure can also be used in wound healing (Figure 6G).

### 6.2 Clinical application of AD‐MSCs in wound healing

The first evidence of ASC use in regenerative medicine was published in 2004. In this case, AD‐MSCs in a fibrin glue in combination with bone grafts were used to treat widespread traumatic calvarial defects in a 7‐year‐old girl who suffered severe head trauma.^212^ Since then, AD‐MSCs have been widely tested for their therapeutic potential in the treatment of numerous diseases. According to ClinicalTrials.gov, 335 clinical trials regarding AD‐MSCs use are registered (https://clinicaltrials.gov/, 12.11.2020, search term: adipose‐derived stem cells) and address a wide range of medical conditions including delayed wound healing, burns, Crohn's disease, diabetes and diabetic wounds, chronic obstructive pulmonary disease, cardiovascular diseases, rheumatoid arthritis, bone and cartilage damage and many others. Interestingly, clinical trials evaluating AD‐MSCs potential in treatment of COVID‐19 are also registered.

To achieve therapeutic potential, AD‐MSCs can be transplanted in various forms: lipoaspirate, SVF, cell suspension of in vitro expanded cells or scaffolded cells. Scaffolds are designed to provide a 3D microenvironment for cell proliferation and differentiation as well as to enhance cell viability, which is beneficial for the regulation of regeneration. Currently much attention is paid to the use of constructs based on AD‐MSCs and scaffolds. Scaffolds should possess several features, such as nontoxicity, nonimmunogenicity, good biodegradability and biocompatibility, and be easy to handle. Additionally, they should also exhibit good chemical and mechanical surface properties (i.e., high porosity) to support cell resistance; promote the adhesion, proliferation, and differentiation of stem cells and allow retention of metabolic futures.^213^, ^214^, ^215^ Clinical trials of fat grafting and SVF application in wound healing are described in Table 5, and clinical trials of AD‐MSCs application in wound healing are summarized in Table 6. Out of 335 identified studies only those specifically regarding the treatment of wounds and additional studies describing the use of fat transfer for wound healing, tissue reconstruction in traumatic injury and oncological patients were summarized. However, another interesting trial—the ACellDREAM II—that is currently recruiting may provide new insight into this topic (NCT03968198). There, Investigators plan to assess the efficacy of use of AD‐MSCs for critical limb ischemia. Inclusion criteria and primary endpoints of the mentioned trial do not require presence of a chronic wounds, as patients only with rest pain and no wounds may be included as well, but this study may provide new evidence on the topic of AD‐MSCs use in wound. Of note, this trial has been processed by a feasibility study (ACellDREAM, NCT01211028) which showed promising results in regard to wound healing.^216^ Clinical trials proved the efficiency of AD‐MSCs in stimulating DFU healing. For example, in a randomized, controlled clinical trial in Korea (clinical trial reg. no.: NCT02619877), the effects of an allogeneic AD‐MSCs sheet (allogeneic AD‐MSCs in a hydrogel, ALLO‐ASCs) were evaluated for the treatment of DFUs. Control patients received treatment with polyurethane film. At Week 8, complete wound closure was observed in 47% and 73% of patients in the control and treatment groups, respectively. At the end of the evaluation (12 weeks) 82% of wounds were completely closed in the group receiving the ALLO‐ASCs sheet, while only 53% were closed in the control group.^217^

**Table 5:** Clinical trials of fat grafting and SVF application in wound healing (clinicaltrials.gov, accessed 12.11.2020)

Study title	Condition/disease	Procedure	Phase	Status	Number of patients	Identification number/Location
3‐D Imaging Assessment of Scar Formation and Would Healing in Fat Grafted vs Nonfat Grafted Facial Reconstruction Wound Sites	Scar formation autologous fat grafting	Autologous fat grafting	Phase 1 Phase 2	Withdrawn	0	NCT01750424/United States
A Study to Evaluate the Results of Facial Soft Tissue Reconstruction in Patients Who Have Suffered Traumatic Injury (BTI)	Facial injuries Adipose tissue	Fat grafting	Not applicable	Completed	20	NCT01345591/United States
Acellular Adipose Tissue (AAT) for Soft Tissue Reconstruction	Soft tissue injuries Trauma	Acellular adipose tissue (AAT)	Phase 2	Active, not recruiting	15	NCT03544632/United States
Adipose Derived Regenerative Cellular Therapy of Chronic Wounds	Diabetic foot Venous ulcer Pressure ulcer	AD‐MSCs	Phase 2	Completed	25	NCT02092870/United States
Autologous Growth Factor Effect on Split‐thickness Donor Site Healing: a Comparison of Adipose Tissue Extract and PRP	Wound healing	Adipose tissue extract Biological: platelet‐rich plasma gel	Phase 2	Completed	24	NCT02799290/Finland
Child's Adipose Cells: Capacity of Tissue Regeneration (cicASChild)	Burns	Adipose tissue sample	Not applicable	Completed	38	NCT02779205/France
Effect of Autologous Fat Grafting on Acute Burn Wound Healing	Burns	Autologous fat grafting Drug: topical cream Procedure: split thickness skin grafting	Phase 3	Recruiting	50	NCT03791710/Egypt
Long Term Status of Free Dermal Fat Autografts for Complex Craniofacial Wounds (FTFDT2)	complex craniofacial wounds	Autologous dermal fat grafting	–	Enrolling by invitation	20	NCT03880188/United States
Platelet Rich Plasma and Autologous Fat Graft for Diabetic Ulcer	DFU	Fat grafting Fat grafting + platelet rich plasma	Not applicable	Unknown	30	NCT03085550/United Kingdom
Safety of Adipose‐Derived Stem Cell Stromal Vascular Fraction	Abnormally healing Wounds Scars Soft tissue defects	ADSC‐ SVF‐002	Phase 1	Not yet recruiting	10	NCT02590042/Canada
Short Term Status of Free Dermal Fat Autografts for Complex Craniofacial Wounds (FTFDT3)	Complex craniofacial wounds	Autologous fat grafting	–	Not yet recruiting	20	NCT03872544/United States
Stromal Vascular Fraction From Lipoaspirate to Treatment of Chronic Non‐healing Wound	Chronic wounds	Antria cell preparation process	Phase 1	Recruiting	40	NCT03882983/United States
Structural Fat Grafting for Craniofacial Trauma: Repeat Fat Grafting Injection‐5 Subject Cohort (BTIPlusUp)	Facial injuries Adipose tissue	Repeat fat grafting	Not applicable	Completed	5	NCT01822301/United States
The Role of Lipoaspirate Injection in the Treatment of Diabetic Lower Extremity Wounds and Venous Stasis Ulcers	Diabetic wounds Venous stasis wounds	Injection of lipoaspirate	Not applicable	Unknown	250	NCT00815217/United States
Treatment of Chronic Leg Ulcers With Autologous Stromal Vascular Fraction	Leg ulcer	Liposuction	Not applicable	Unknown	30	NCT02987101/Denmark
		Standard wound care				
		Adipose‐derived regenerative cells				
Treatment of Hypertensive Leg Ulcer by Adipose Tissue Grafting (Angiolipo)	Skin ulcer	Adipose tissue grafting	Not applicable	Unknown	10	NCT01932021/France
Use of Concentrated Endogenous Autologous Adipose Stromal Cells in Fat Grafts for Craniofacial Trauma (ARM5)	Craniofacial injuries	Fat grafting	Not applicable	Terminated	5	NCT01633892/United States
Healing Chronic Venous Stasis Wounds With Autologous Cell Therapy	Nonpenetrating wound	Autologous SVF	Not applicable	Active, notrecruiting	36	NCT02961699/United States
		Device: transpose RT System				
		Other: debridement/dressing of wound				
Assessment of the Efficacy and Tolerance of Subcutaneous Reinjection of Autologous Adipose‐derived Regenerative Cells in the Local Treatment of Neuropathic DFUs	DFU	Drug: adipose derived regenerative cells (as SVF)	Phase 2	Unknown	45	NCT02866565/France
Nanofat on Wound Healing and Scar Formation	Scars	Nanofat injection (lipoaspirate)	Not applicable	Not yet recruiting	15	NCT03850119/Belgium
	Delayed Wound Healing					
	Hypertrophic Scar					
	Postinflammatory					
	Hyperpigmentation					
	Donor site complication					
Adipose‐Derived Stromal Cells (ASC's) for Pressure Ulcers	Pressure ulcer	Biological: adipose derived stromal cells (SVF)	Phase 1	Active, not recruiting	12	NCT02375802/United States
Autologous Adipose derived Regenerative Cells Injection for Treatment of Radiation‐induced Rectovaginal Fistula	Rectovaginal fistula	Injection of autologous regenerative cells of adipose tissue (cells obtained by enzymatic digestion of lipoaspirate)	Phase 1	Completed	16	NCT03643614/Russian Federation
19F Hot Spot MRI of Human Adipose‐derived Stem Cells for Breast Reconstruction	Breast cancer	Drug: CS‐1000 labeled SVF cells	Phase 1	Recruiting	6	NCT02035085/United States
Pilot Study of Skin Quality Improvement After AD‐MSCs Transfer in Irradiated Breasts	Breast neoplasms	Biological: adipose SVF cell	Not applicable	Not yet recruiting	10	NCT01801878/Republic of Korea
	Skin abnormalities	Biological: normal saline				
Study of Autologous Fat Enhanced w/Regenerative Cells Transplanted to Reconstruct Breast Deformities After Lumpectomy	Breast neoplasms	ADRC enhanced autologous	Phase 4	Completed	71	NCT00616135/Belgium, Italy, Spain, United Kingdom
	Carcinoma, ductal, breast					
	Mammaplasty	Fat transplant				
	Mastectomy, segmental, lumpectomy, breast reconstruction					
Autologous Fat Transfer for Scar Prevention and Remodeling	Wound	Autologous fat transfer	Phase 1 Phase 2	Completed	14	NCT01119326/United States
Effect of Concentrating Endogenous Stromal Cells in the Fat Graft	Facial injuries	Fat graft surgical procedure	–	Terminated	3	NCT01564524/United States
	Adipose tssue					
Effect of Concentrating Endogenous Stromal Cells in the Fat Graft Using TGI Device	Facial injuries	Device: tissue genesis CellIsolation System (TGI CIS)	Not applicable	Completed	7	NCT01924364/United States
	Tissue injury	Procedure: standard of carefat grafting				
Fat Grafting in Skin‐grafted Deep Burn Scars	Burn scar	Procedure: lipofilling/fatgrafting	Not applicable	Completed	15	NCT03627650
		Procedure: placebo injection				
Structural Fat Grafting for Craniofacial Trauma Using Manual Technique for Processing Fat Graft Material	Facial injuries	Procedure: fat grafting	Not applicable	Completed	15	NCT02267187/United States
		Drug: general anesthesia				
		Device: Coleman cannulas				
		Other: Tefla nonadherent gauze pad				
Adipose‐Induced Regeneration of Breast Skin to Treat Postmastectomy Radiation Injury in Breast Cancer Patients	Breast cancer	Fat grafting	Not applicable	Not yet recruiting	20	NCT03981718/United States
Autologous Adipose Tissue in the Treatment of Systemic Sclerosis Digital Ulcers	Systemic sclerosis	Procedure: autologous fat grafting	Not applicable	Unknown	46	NCT03406988/Italy
	Digital ulcer	Procedure: Sham procedure				
Autologous Micro‐fragmented Adipose Tissue in the Treatment of Minor Amputations of Diabetic Foot	Diabetic foot	Device: lipogems	Not applicable	Completed	112	NCT03276312/Italy
Comparative Study Between Fat Injection And Platelet Rich Plasma In Post Burn Facial Scar	Burn scar	Platelet rich plasma injection in post burn facial scar	Not applicable	Not yet recruiting	60	NCT04557514
		Fat injection in post burn facial scar				

**Table 6:** Clinical trials of AD‐MSCs application in wound healing (clinicaltrials.gov, accessed 12.11.2020)

Study title	Condition/disease	Procedure	Phase	Status	Number of patients	Identification number
Treatment of Chronic Wounds in Diabetic Foot Syndrome With Autologous Adipose Derived MSCs	DFU	Biological: application of autologous ADSC stem cells in fibrin gel Procedure: standard care in DFU	Phase 1 Phase 2	Recruiting	20	NCT03865394/Poland
Autologous Keratinocyte Suspension Versus ASC‐Keratinocyte Suspension for Postburn Raw Area	Burn with full‐thickness skin loss	Procedure: noncultured autologous keratinocyte suspension Procedure: ASC‐keratinocyte suspension Procedure: split skin graft	Not applicable	Not yet recuiting	33	NCT03686449/Egypt
A Follow‐up Study to Evaluate the Safety of ALLO‐ASC‐DFU in ALLO‐ASC‐BI‐101 Clinical Trial	Burn	Biological: ALLOASC‐ DFU	–	Completed	5	NCT03183622/Republic of Korea
Allogeneic ADSCs and Platelet‐ Poor Plasma Fibrin Hydrogel to Treat the Patients With Burn Wounds (ADSCs‐BWs)	Second‐ or third‐degree Burns	Biological: ALLOASCs	Phase 1 Phase 2	Unknown status	20	NCT03113747/Ukraine
A Study to Evaluate the Safety of ALLO‐ASC‐DFU in the Subjects With Deep Second degree Burn Wound	Burn	Biological: ALLOASC‐DFU	Phase 1	Completed	5	NCT02394873/Republic of Korea
Clinical Study of AD‐MSCs in the Treatment of Diabetic Foot	DFU	Biological: MSCs treatment	Phase 1	Not yet recriuting	60	NCT03916211/China
Clinical Study of ALLO‐ASC SHEET in Subjects With DFUs	DFU	Biological: ALLOASC‐DFU Procedure: hydrogel SHEET (vehicle control)	Phase 2	Recriuting	44	NCT03754465/United States
Clinical Study to Evaluate Efficacy and Safety of ALLOASC‐ DFU in Patients With DFUs	DFU	Biological: ALLOASC‐DFU Procedure: vehicle sheet	Phase 3	Unknown status	164	NCT03370874/Republic of Korea
A Follow‐up Study to Evaluate the Safety of ALLO‐ASC‐DFU in ALLO‐ASC‐DFU‐101 Clinical Trial	DFU	Biological: ALLOASC‐DFU	–	Completed	4	NCT03183726
A Clinical Study Using ASCs for Diabetic Foot	Peripheral vascular diseasei	Biological: AD‐MSCs	Phase 1	Unknown status	240	NCT02831075/China
	Ischemia					
	Diabetic foot	Biological: saline				
Safety of ALLO‐ASC‐DFU in the Patients With DFUs	DFU	Biological: ALLOASC‐ DFU	Phase 1	Completed	5	NCT02394886/Republic of Korea
A Follow‐up Study to Evaluate the Safety of ALLO‐ASC‐DFU in ALLO‐ASC‐BI‐201 Clinical Trial	Burn	Biological: ALLOASC‐DFU	–	Enrolling by invitation	30	NCT03183648/Republic of Korea
A Follow‐up Study to Evaluate the Efficacy and Safety for the Patients With ALLO‐ASC‐DFU Treatment in Phase 1/2 Clinical Trial of ALLO‐ASC‐EB‐101	Dystrophic epidermolysis bullosa	Biological: ALLO‐ASC‐DFU	–	Not yet recruiting	5	NCT03183934/Republic of Korea
Clinical Application of Mesenchymal Stem Cells Seeded in Chitosan Scaffold for Diabetic Foot Ulcers	Stem cell transplant	Drug: stem cell product	Phase 1	Unknown	40	NCT03259217
Subcutaneous Injections of Autologous ASC to Heal Digital Ulcers in Patients With Scleroderma.	Systemic slerosis	Procedure: adipose tissue harvest	Phase 2	Recruiting	32	NCT04356755/France
		Drug: autologous ASC				
		Drug: Placebo				
Treatment of Patients With Trophic Ulcers Using Mesenchymal Stem Cells	Trophic ulcer	Biological: autologous adipose‐derived mesenchymal stem cells	Phase 1 Phase 2	Completed	18	NCT04457037/Belarus
Safety and Efficacy of Allogeneic Adipose Tissue Mesenchymal Stem Cells in Diabetic Patients With Critical Limb Ischemia	Limb ischemia	Drug: high dose allogeneic mesenchymal stromal cells	Phase 2	Not yet recruiting	90	NCT04466007
	Diabetic foot	Drug: low dose allogeneic mesenchymal stromal cells				
		Drug: placebos				
Clinical Study of ALLO‐ASC‐SHEET in Subjects With Diabetic Wagner Grade II Foot Ulcers	DFU	Biological: ALLO‐ASC SHEET	Phase 2	Not yet recruiting	64	NCT04497805
Clinical Study to Evaluate Efficacy and Safety of ALLO‐ASC‐DFU in Patients With Diabetic Wagner Grade 2 Foot Ulcers	DFU	Biological: ALLO‐ASC‐DFU	Phase 3	Recruiting	104	NCT04569409/Republic of Korea
		Procedure: vehicle sheet				

Attempts to transplant AD‐MSCs into radiotherapy‐ or cancer‐damaged tissues were also made. For example, fat transfer followed by split‐thickness skin grafting was performed in a 67‐year‐old woman with a chronic, nonhealing ulcer on her leg resulting from squamous cell carcinoma excision and radiotherapy, this resulted in complete healing of the ulcer.^195^ Fat transfer was also used in patients after radiotherapy for breast or head and neck cancers (Table 7).

**Table 7:** Examples of AD‐MSCs therapy in patients with skin complications after oncological treatment

	Procedure	Number of patients	Results	Reference
Effects of lipofilling on the functional and the esthetic aspects of irradiated breast reconstruction	Serial autologous fat grafting	Study group: 20 patients Control group: 41 patients (42 breasts)	Significant improvement in LENT‐SOMA scores after treatment compared to control and those before treatment Significantly enhanced cosmetic outcomes Implant exposure in two cases in the active branch with severe flap thinning in control group, while in study Group 4 cases with this problem resulted with no implant exposure	Panettiere et al.^218^
Lipofilling on irradiated expanders in patients undergoing postmastectomy radiotherapy	Autologous fat grafting by Coleman technique	Study group: 16 patients Control group: 16 patients	No ulceration and implant exposure in the irradiated area in the study group compared to 31.25% extrusion rate of the implant in control group The shape and symmetry were significantly better in study group	Ribuffo et al.^219^
Lipoaspirate transplant into tissue damaged by radiotherapy	Autologous transplantation of purified lipoaspirate	20 Patients tissue damage after radiotherapy for breast cancer— LENT‐SOMA Grade 3 (severe symptoms) or Grade 4 (irreversible functional damage)	Profound improvement of symptoms in 19 of 20 patients Progressive regeneration (neovessel formation and improved hydration) observed in tissue ultrastructure	Rigotti et al.^220^
Fat grafting in irradiated head and neck tissues	Autologous fat transplants similar to Coleman technique	11 Patients in cancer remission after radiotherapy requiring aesthetic subcutaneous or submucous head and neck reconstruction	Esthetic and functional rehabilitation in 10 of 11 patients Improvement in patients’ quality of life Normal histologic structure, absence of necrotic areas and a highly dense vascular network	Phulpin et al.^221^

Abbreviation: AD‐MSC, adipose‐derived mesenchymal stromal cell.

### 6.3 Allogeneic versus autologous therapy and the immunological properties of AD‐MSCs

The use of allogeneic cells gives rise to the possibility of rapid application of cells to the patient and enables full cell characterization before therapy. Additionally, a small number of donors would provide treatment for many patients; hence, immunogenicity tests and analyses of transplantation safety in allogeneic systems are very important. Allogeneic transplants allow the pooling of cells from many donors and standardization of the cell product.^222^ The application of allogeneic, stored cells is already in use. However, there are still some issue to be considered, for example, a lack of standardized parameters for handling frozen cells. Another issue is the lack of uniform guidelines for all AD‐MSCs banking processes, such as donor recruitment, manipulation, banking procedures, exemption and specific postthaw qualification tests. It would ensure multiple treatment of the patients, avoiding recurrent liposuction.^223^, ^224^

AD‐MSCs are suitable candidates for allogeneic cell therapies due to their low immunogenic profile, which was demonstrated by low expression of major histocompatibility complex (MHC) Class II molecules, and T and B cell costimulatory molecules CD80, CD86, and CD40 in vitro.^225^ However, in vivo studies have shown that AD‐MSCs can induce a humoral and cellular immune response; hence, they are not fully immunologically privileged cells. AD‐MSCs may also be under the influence of inflammation‐based signaling and induce the expression of MHC II molecules and Toll‐like receptors. In addition, AD‐MSCs may also increase first‐ and second‐class MHC expression as a result of differentiation.^226^, ^227^, ^228^ *In vitro* cell culture can also influence immunogenicity; for example, cell culture with bovine serum slightly reduces cell immunogenicity compared to that in culture without serum.^229^

Following modulation of the local inflammatory environment, it is believed that the final determination of immunomodulatory responses is likely elicited by the differentiation of cells, a combined action of direct cell–cell contacts and the secretion of soluble factors.^229^, ^230^ The immunological properties of AD‐MSCs are also dependent on the donor's concomitant diseases. Cells derived from obese Type 2 diabetes (T2D) patients show increased expression of inflammatory markers, activation of the NLRP3 inflammasome, and a greater capacity for migration and phagocytosis than those derived from lean donors. Interestingly, AD‐MSCs derived from obese and T2D individuals also show a reduction in typical immunosuppressive activities attributed to MSCs.^231^ Moreover, it has recently been proven that Crohn's disease disturbs the immune activities of AD‐MSCs, which is related to inflammasome activation.^232^

## 7 MSCs INTERACTIONS WITH IMMUNE CELLS

It should be noted that MSCs also take part in regenerative processes by affecting the immune system. MSCs cells produce number of modulating and immunosuppressive cytokines as well as they can affect immune cells through direct interactions.^233^, ^234^ MSCs may inhibit the activity of natural killer (NK) cells, lymphocytes Tc and dendritic cells (DCs).^235^ MSCs can also induce the transition of M1 macrophages into M2 macrophages, thus promoting tissue regeneration. M2 produce various cytokines and growth factors such as IL‐10, TGF‐β1, PDGF, IGF‐1, and VEGF, which may stimulate cell proliferation, granulation tissue formation, angiogenesis and wound healing.^7^, ^8^ One of the immunosuppressive mechanisms of MSCs activity is also the production of indoleamine 2,3‐dioxygenase, which is involved in the l‐tryptophan catabolism. This process leads to a reduction in the level of tryptophan in the microenvironment at the same time increasing the level of kynurenine which inhibits the activity of T cells, NK cells, and DCs.^236^ MSCs also use other immunosuppressive and antiinflammatory agents such as nitric oxide synthase (iNOS), TNF‐α‐stimulated gene‐6 (TSG6) and prostaglandin E2 (PGE2). MSCs have been shown to increase the expression of iNOS in response to proinflammatory cytokines (TNF‐α, IL‐1) and IFN‐γ.^237^, ^238^ Another factor produced by MSCs is a glycoprotein TSG‐6 (35 kDa). It is believed that TSG‐6 counteract inflammatory effects of TNF and IL‐1 thus inhibiting inflammation. Interestingly, proper expression of the TSG‐6 is crucial to achieve the therapeutic effect of MSC cells.^239^ PGE2 is also involved in modulating the activity of the immune system by inhibiting the inflammatory response and stimulating Treg lymphocytes.^233^, ^240^

## 8 STRATEGIES FOR STIMULATING OF AD‐MSCs ACTIVITIES IN WOUND HEALING

Numerous clinical trials investigating the therapeutic potential of autologous or allogenic AD‐MSCs in the treatment of different diseases have been conducted. However, their results are unsatisfactory, which creates a need to search for new strategies of AD‐MSCs stimulation to enhance their activity and translate AD‐MSCs‐based therapies to everyday clinical practice. Several strategies, including preconditioning with bioactive molecules, genetic engineering of AD‐MSCs, modification of culture conditions and direct application of exosomes or extracellular vesicles, were established to overcome these problems. The impact of these strategies on improving of AD‐MSCs potential in wound healing was evaluated in in vitro and in vivo experiments.^241^

It was shown that AD‐MSCs cultured in endothelial growth medium enhance their proangiogenic properties, which may increase their therapeutic potential in ischemic diseases, for example, ischemic wounds.^242^ In another study, AD‐MSCs administrated with bFGF via sustained release from a gelatin hydrogel showed increased secretion of angiogenic growth factors and vessel maturation in a murine ischemic hind limb model.^243^ Moreover, priming of AD‐MSCs with deferoxamine upregulated VEGF expression in a time and dose‐dependent manner, which was mediated by augmentation of HIF‐1 activity in AD‐MSCs, and increased their paracrine effect on endothelial cells (i.e., increase in migration).^244^ PDGF‐D can also be used for AD‐MSCs preconditioning before transplantation because it increases their proliferation and migration; upregulates expression of growth factors (such as VEGFA, FGF1, FGF5, bone morphogenetic protein 8B [BMP8B], leukemia inhibitory factor [LIF], inhibin beta A [INHBA], IL11, and heparin‐binding EGF‐like growth factor [HBEGF]) in AD‐MSCs and enhances their regenerative potential.^245^

The preconditioning of AD‐MSCs and modifications of other culture conditions can affect the effectiveness of AD‐MSCs in wound healing. In traditional 2D cell culture cells are grown in a monolayer; however, this method does not fully reflect conditions prevailing in normal tissue, such as cell‐to‐cell or extracellular interactions. In recent years, 3D spheroid cell culture was developed, which better reflects these interactions and can be helpful in improving of AD‐MSCs the therapeutic potential.^246^ Cheng et al.^247^ cultured AD‐MSCs in spheroids and then expanded them in monolayers to determine whether they exhibit superior pro‐regenerative activity. They found that compared to 2D‐cultured AD‐MSCs, spheroid‐cultured AD‐MSCs show higher expansion, less senescence, higher levels of pluripotency markers, C‐X‐C chemokine receptor type 4 (CXCR4) and angiogenic growth factors and enhanced migration and expression of MMP‐9 and MMP‐13. Moreover, in a murine model of impaired wound healing, the spheroid‐cultured cells caused faster healing and increased angiogenesis with better cell engraftment in the wound as well as evidence of their differentiation into epidermal and endothelial lineages. Amos et al.^248^ showed that AD‐MSCs formulated in multicellular aggregates had better acceleration of diabetic wound closure than did an equal number of cells delivered via suspension. In addition, AD‐MSCs cultured in hypoxic conditions were shown to have enhanced proregenerative potential owing to their increased proliferation rate and secretion of growth factors (VEGF, bFGF). It was also proved that hypoxia upregulated VEGF and bFGF expression on messenger RNA and protein level. Additionally, the conditioned medium from AD‐MSCs cultured in hypoxic conditions compared to normoxia‐cultured AD‐MSCs conditioned medium, stimulated collagen synthesis and migration of human dermal fibroblasts as well as accelerated wound closure in a mice full‐thickness wound model.^249^ Culturing MSCs under hypoxic conditions also helps to improve their self‐renewal capacity and retain undifferentiated phenotype.^250^

Genetic modifications were also shown to increase AD‐MSCs activity in wound healing. Kim et al.^251^ proved that overexpression of CXCR4 in AD‐MSCs leads to an increase in their homing and engraftment into ischemic areas after transplantation. These cells also promoted long‐term engraftment and muscle tissue regeneration in diabetic mice with hindlimb ischemia. Similarly, AD‐MSCs with gene transfer of manganese superoxide dismutase (SOD2) that were transplanted into syngeneic mice elicited cytoprotective effects and improved survival and engraftment rates.^252^ Additionally, overexpression of Oct4 and Sox2 in AD‐MSCs increases their proliferation and differentiation and can be helpful in expanding and enhancing their stemness.^253^

Direct application of AD‐MSCs‐derived exosomes may also have therapeutic potential in cutaneous wound healing. Conducted studies revealed that their application accelerates wound healing in a murine model by optimizing fibroblast functions. AD‐MSCs exosomes stimulate the production of collagen I and III in the early stages of wound healing and reduce scar formation by decreasing collagen expression in the late stages.^254^ In a continued study, it was found that AD‐MSCs‐derived exosomes promote scarless wound healing through regulation of ECM remodeling (increases in TGFβ3:TGFβ1 and MMP3:TIMP1 ratios and prevention of myofibroblast differentiation), and the mechanism involves activation of the ERK/MAPK pathway.^255^ It was also demonstrated that stimulation with inflammatory cytokines (TNF‐α/IFN‐γ) can enhance the immunosuppressive and antiinflammatory potential of AD‐MSCs‐derived exosomes.^256^

## 9 CONCLUSIONS

AD‐MSCs seem to be a promising tool for the treatment of various types of diseases. They are successfully used, among others, in the treatment of chronic or complicated wounds of various etiologies, for example, diabetic wounds. These cells secrete a number of cytokines and growth factors that stimulate the processes responsible for proper wound healing. They are also a good model for testing the activity of new proregenerative compounds. However, despite the large number of studies on the biology and characterization of AD‐MSCs and the existence of clinical trials evaluating their therapeutic potential, the topic of their safety is still widely discussed, particularly in relation to cancer patients. There is still a lack of an adequate number of clinical trials on the effectiveness of AD‐MSCs in the treatment of oncological wounds. The number of complicated wounds and tissue defects arising as a result of cancer and oncological treatment is a major problem in the daily work of oncologists, these adverse event significantly worsen the patients' quality of life. However, it seems that these cells should be used with extreme caution in cancer patients, once the patient is made aware of the risk. The issue of their safety and effectiveness in stimulating wound healing in cancer patients should certainly be further investigated and relevant clinical trials should be performed. It is also worth mentioning that some limitations of cellular therapies are their high costs, restrictive legal regulations and the time needed for cell expansion. In conclusion, the applications of these cells are very attractive, but they still require more basic and clinical investigations.

## AUTHOR CONTRIBUTIONS

Milena Deptuła: participated in manuscript conceptualization, drafted the manuscript, described wound healing process of acute and chronic wounds including wound healing in diabetes and oncological patients, AD‐MSCs characteristics (markers, differentiation, genetic stability, effect of FBS deprivation), AD‐MSCs role in wound healing and their clinical application (Tables 6 and 7) and strategies of stimulation of their activities, prepared figures, conducted part of the clinical trials research, revised the manuscript; Agnieszka Brzezicka: provided photos of fat grafting by Coleman technique, conducted part of clinical trials research, described Coleman technique and prepared Table 1; Aneta Skoniecka: provided photos of cell isolation, described cell isolation and cells and tissue sources, compared SAT and VAT (with Table 4), compared AD‐MSCs and SVF (with Table 2) and described factors affecting AD‐MSCs and BAT and WAT AD‐MSCs; Jacek Zieliński: provided photos of fat harvesting through surgical resection and wound healing complications in oncological patients and critically revised the manuscript; Michał Pikuła: participated in manuscript conceptualization, described immunological properties of AD‐MSCs, MSCs interactions with immune cells, supervised work, critically revised the manuscript and provided financial support.

All authors approved final version of the manuscript. The authors declare that there are no conflict of interests.
